# Supplementary material for: Association between oral microbiome and seven types of cancers in East Asian population: a two-sample Mendelian randomization analysis
Source: Front Mol Biosci. 2023 Nov 21;10:1327893. doi: 10.3389/fmolb.2023.1327893 (PMC10702768; doi:10.3389/fmolb.2023.1327893)
Supplement: Supplementary file 1 [file DataSheet1.ZIP › Supplementary Materials/MR plots of saliva/saliva═╝/ovarian cancer/pheno.3563_to_ovarian cancer_leave_one_out.pdf]

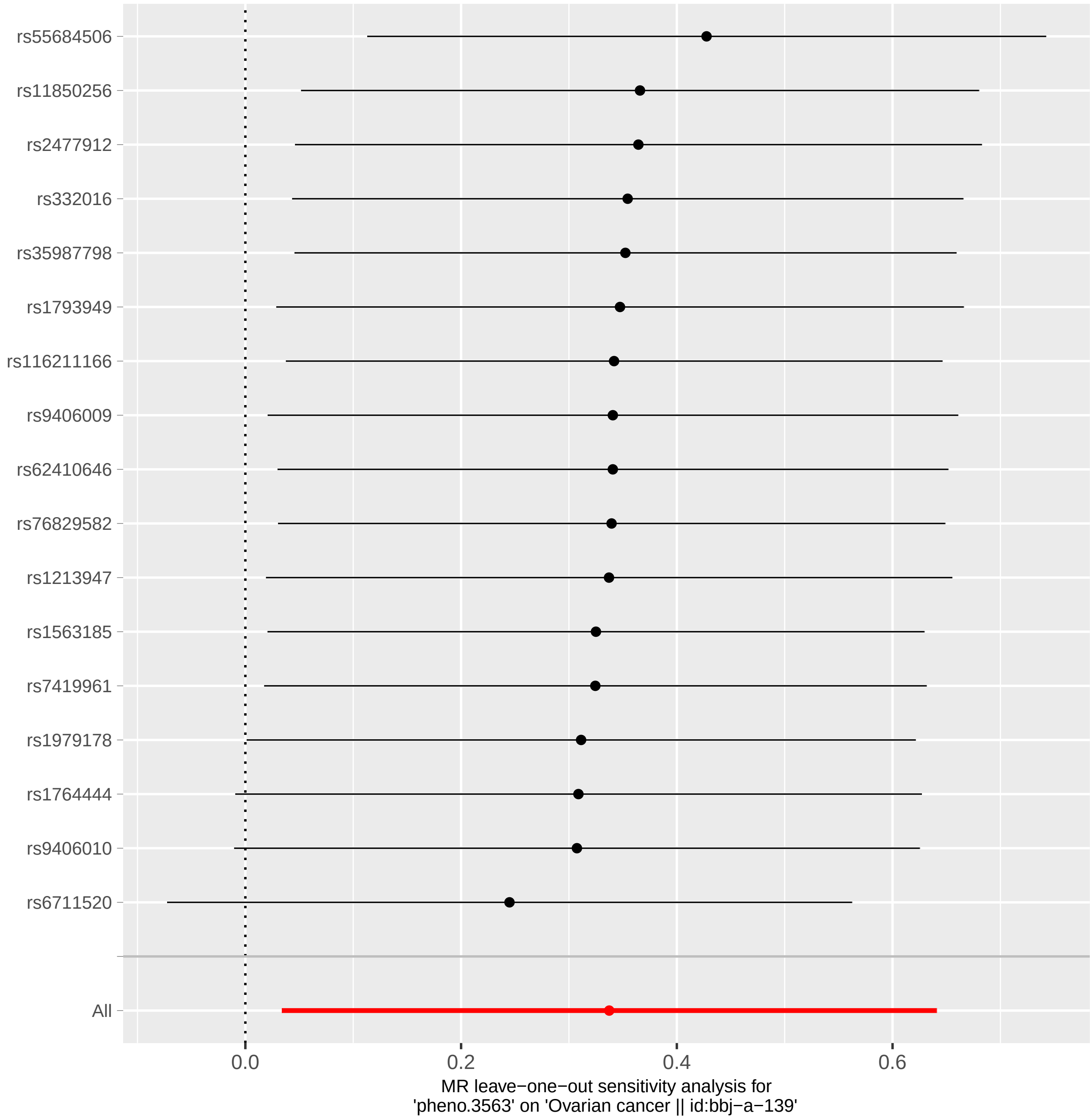
rs55684506
rs11850256
rs2477912
rs332016
rs35987798
rs1793949
rs116211166
rs9406009
rs62410646
rs76829582
rs1213947
rs1563185
rs7419961
rs1979178
rs1764444
rs9406010
rs6711520
All
0.0
0.2
0.4
0.6
MR leave−one−out sensitivity analysis for
'pheno.3563' on 'Ovarian cancer || id:bbj−a−139'
